# Study on the correlation between iris blood flow, iris thickness and pupil diameter in the resting state and after pharmacological mydriasis in patients with diabetes mellitus

**DOI:** 10.1186/s12886-024-03322-y

**Published:** 2024-02-02

**Authors:** Lipu Cui, Ying Xiao, Zhaoyu Xiang, Zhangling Chen, Chenhao Yang, Haidong Zou

**Affiliations:** 1 Department of Ophthalmology, Shanghai General Hospital, Shanghai Jiao Tong University School of medicine, Shanghai, China; 2 Shanghai Eye Diseases Prevention & Treatment Center, Shanghai Eye Hospital, Shanghai, China; 3 Department of Ophthalmology, Children’s Hospital of Fudan University, No. 399 Wanyuan Road, 201102 Shanghai, China; 4 Department of Ophthalmology, Shanghai Songjiang District Central Hospital, Shanghai, China; 5 Shanghai Key Laboratory of Fundus Diseases, Shanghai, China; 6 National Clinical Research Center for Eye Diseases, Shanghai, China; 7 Shanghai Engineering Center for Precise Diagnosis and Treatment of Eye Diseases, Shanghai, China

**Keywords:** iris blood flow, iris thickness, Type 1 diabetes, Type 2 diabetes, Pupil abnormalities

## Abstract

**Background:**

To investigate whether iris blood flow and iris thickness at the iris smooth muscle region affect the pupil diameter at rest and after drug-induced mydriasis in patients with type 1 diabetes mellitus (T1DM) and type 2 diabetes mellitus (T2DM).

**Methods:**

T1DM patients and healthy children were recruited from the SCADE cohort. T2DM patients and healthy adults were recruited from patients undergoing cataract surgery at Shanghai General Hospital. Iris vessel density, pupil diameter (PD) and iris thickness were measured in both the resting and drug-induced mydriasis states. Iris vessel density was measured by optical coherence tomography angiography (OCTA), PD was measured by a pupilometer, and iris thickness at the iris smooth muscle regions were measured using anterior segment optical coherence tomography (AS-OCT).

**Results:**

The study included 34 pediatric T1DM patients and 50 adult T2DM patients, both groups without diabetic retinopathy, and age-sex-matched healthy controls. At baseline, T1DM children and healthy children showed no differences in iris blood flow, iris thickness, or PD. However, the adult T2DM group exhibited higher vessel density at the pupil margin, thinner iris thickness at the iris dilator region, and smaller PD compared to healthy adults, with these differences being statistically significant (*P* < 0.05). After pupil dilation, there were no changes in iris blood flow and PD in the T1DM group compared to healthy children, whereas the T2DM group showed a significantly smaller PD compared to healthy adults. Multivariate regression analysis revealed that in the T2DM group, glycated hemoglobin was an independent factor of PD after dilation (β=-0.490, *p* = 0.031), with no such factors identified in the T1DM group.

**Conclusion:**

The insufficiently dilated pupil diameter after drug-induced mydriasis is correlated to the level of glycated hemoglobin among T2DM patients.

**Trial registration:**

The registration number on the clinical trial website was NCT03631108.

## Introduction

Pupil dilation is an important procedure in ophthalmology for screening and treating retinal diseases [1]. Insufficient pupil dilation reduces the sensitivity of retinal disease screening; increases the difficulty of cataract, retinal laser photocoagulation and other surgical procedures; and increases the risk of iatrogenic injury [2, 3]. Since the 1980s, researchers have discovered abnormal pupil size in type 1 and type 2 diabetes patients [4–6]. In both the resting state and drug-induced mydriasis, the pupil diameter of diabetes patients is smaller than that of healthy controls. The exact mechanism is not yet clear, and several possible reasons are currently under speculation. First, due to autonomic neuropathy in diabetes [7], neuromuscular effectors may be damaged, and synapse loss may occur, which affects the sympathetic and parasympathetic nerves’ control of the smooth muscles of the iris. Ishikawa [8] observed mitochondrial abnormalities, dense bodies, and layered structures under an electron microscope in the peripheral nerves of the iris dilator and sphincter muscles in diabetic patients. Second, the advanced glycosylation end products of diabetes metabolism may cause damage to the smooth muscles of the iris, and the muscles’ function and contraction strength could be severely affected. Electron microscopy also showed lipid droplets and vacuolar changes in the smooth muscle fibers of the iris of diabetes patients [8]. Currently, there is no imaging technology to directly observe autonomic nerves and iris smooth muscle abnormalities in living diabetes patients’ eyes. Current anterior OCT technology cannot distinguish iris smooth muscles from other tissues, but researchers can indirectly evaluate iris muscle condition through iris thickness at the iris sphincter and dilator regions. For example, Prata et al. successfully found imaging evidence of iris muscle alterations caused by α-blockers through anterior OCT technology [9]. There is no relevant research on iris thickness at the iris sphincter and dilator regions through anterior OCT in diabetic eyes.

High blood sugar not only affects the nervous and muscular systems but also invades blood vessels [10, 11]. The blood circulation in the iris, as the only vascular system in the anterior chamber, plays an obvious role in providing nutrition for the iris muscles and nerves. However, the potential role of iris vascular lesions among diabetic patients in pupil abnormalities has not been well explored for many years. In previous studies by our research group, using self-developed iris OCTA quantitative analysis technology, significant differences in iris blood flow between type 2 diabetes patients and healthy individuals have been observed [12]. Based on this, we speculate that iris vascular lesions or reduced blood flow may promote iris smooth muscle damage and peripheral nerve damage, leading to smaller pupils in diabetic patients. In this study, we observed pediatric type 1 diabetes patients and adult type 2 diabetes patients using iris OCTA quantitative analysis technology combined with AS-OCT to measure iris thickness at the iris sphincter and dilator muscle regions. We analyzed whether changes in these indicators were related to pupil diameter at rest or after drug-induced mydriasis, aiming to explore the mechanism of insufficient drug-induced mydriasis in diabetic patients and provide new ideas for addressing this problem.

## Materials and methods

This was a prospective cross-sectional study that included two groups of participants: [1] Pediatric T1DM patients and healthy children were members of the Shanghai Children and Adolescent Diabetes Eye (SCADE) cohort and attended Shanghai Eye Hospital in February 2022. SCADE is a diabetic study cohort followed up each year since 2018, with research results on retinal thickness and retinal blood flow already reported [2]. Adult T2DM patients and healthy adults were patients who visited Shanghai General Hospital Affiliated with Shanghai Jiaotong University School of Medicine from October 2021 to September 2022. This study conformed to the guidelines presented in the Declaration of Helsinki. Written informed consent was obtained from all participants or their guardians. The adolescent participants’ examinations were performed with their guardians in attendance. This study was approved by the ethics committee of Shanghai General Hospital (approval numbers 2018KY209 and 2018KY181). The registration number on the clinical trial website was NCT03631108.

The inclusion criteria were as follows: [1] children and adolescents diagnosed with T1DM or adults diagnosed with T2DM according to the World Health Organization diagnostic criteria (diabetic group) and healthy children matched for age, sex, and axial length and healthy adults matched for age, sex, and axial length (healthy control group); [2] participants who had stable fixation and could cooperate with all the examinations in the study, including iris OCTA and AS-OCT examinations; [3] best-corrected visual acuity better than 20/25 for pediatric patients or better than 20/50 for adult subjects, who were all cataract patients; and [4] participants who signed an informed consent form. The exclusion criteria were as follows: [1] diagnosis of diabetes retinopathy (DR, based on the international DM classification criteria) [10, 11]; [2] presence of eye diseases that may hinder OCTA examination, such as eyelid disease or strabismus; [3] presence of eye diseases that may induce iris vasculature alterations, such as retinal vein occlusion; [4] presence of obvious iris changes, such as tumors, nodules and neovascularization; [5] history of eye injuries or surgeries; [6] recent use of eyedrops within the last month that could influence pupil diameter, such as atropine or pilocarpine, excluding those used for routine rapid pupil dilation; and [7] presence of certain neurological disorders may impact the nerve supply to the iris and the extent of pupil dilation, excluding diabetes and hypertension.

All participants underwent systemic and ophthalmic examinations. All examiners were masked to the group information of participants, whether they belonged to the diabetic group or the healthy control group. Systemic examinations included height, weight, and systolic and diastolic blood pressure. The plasma glycated hemoglobin level of diabetic patients was measured. Eye examinations included the following: [1] best-corrected visual acuity measured by the international standard LogMAR visual acuity chart and refraction measured using a computerized automatic optometry machine (KR-8900, Topcon, JAPAN); [2] examinations of the eyelid, conjunctiva, cornea, anterior segment, iris, pupil, and lens performed with a slit lamp (SL130, Zeiss, Germany) and fundus examinations performed with a noncontact lens (90D, Ocular, US); [3] axial length measured with an IOL Master 700 (Carl Zeiss Meditec, Dublin, CA); [4] intraocular pressure examined with a noncontact tonometer (NT-530P, NIDEK, Tokyo, JAPAN); [5] examination of the posterior fundus using SSOCT (Triton, Topcon, Tokyo, JAPAN); [6] measurements of pupil diameter after adaptation to ambient light for 5 min using a handheld pupilometer (PLR3000, NeurOptics, California, USA), where the final result was the average value of five measurements; [7] iris OCTA examination (Cirrus HD-OCT using a 3 mm*3 mm scan mode); and [8] examination of the anterior segment and iris volume and iris thickness at the dilator pupillae region and sphincter pupillae region using AS-OCT (SS-1000, CASIA, Tomey, Nagoya, JAPAN) in 3D scan mode. The testing room for children had an illuminance of 300 lx, while the testing room for adults had an illuminance of 150 lx. After a 5-minute adaptation period, baseline measurements were taken in the undilated state, and the measurements for drug-induced mydriasis were obtained at full achievement of pupil dilation. Children received 1% cyclopentolate (Cyclogyl, Alcon, US), while adults received 0.5%tropicamide and 0.5% phenylephrine eye drops (Mydrin-P, Santen, US). Eye drops were applied to the conjunctival sac at five-minute intervals, a total of three times, until the pupil achieved full dilation. Once fully dilated, the condition was monitored and maintained stable for a duration of 20 min.

**Fig. 1:**
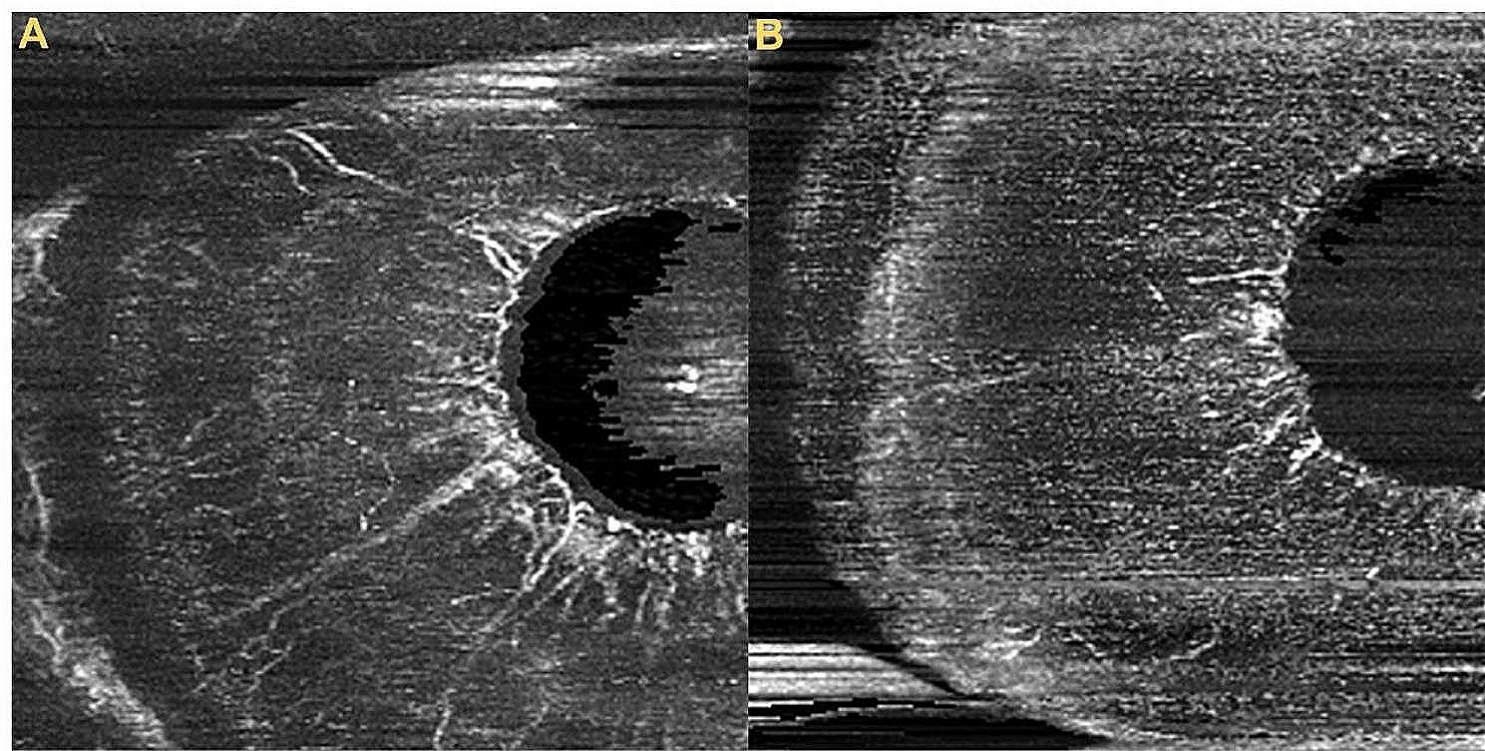
Picture **A** was an OCTA image of a type 2 diabetes adult, the pupillary VSD was 0.28. Picture **B** was an OCTA image of a healthy adult, the pupillary VSD was 0.26.

**Fig. 2:**
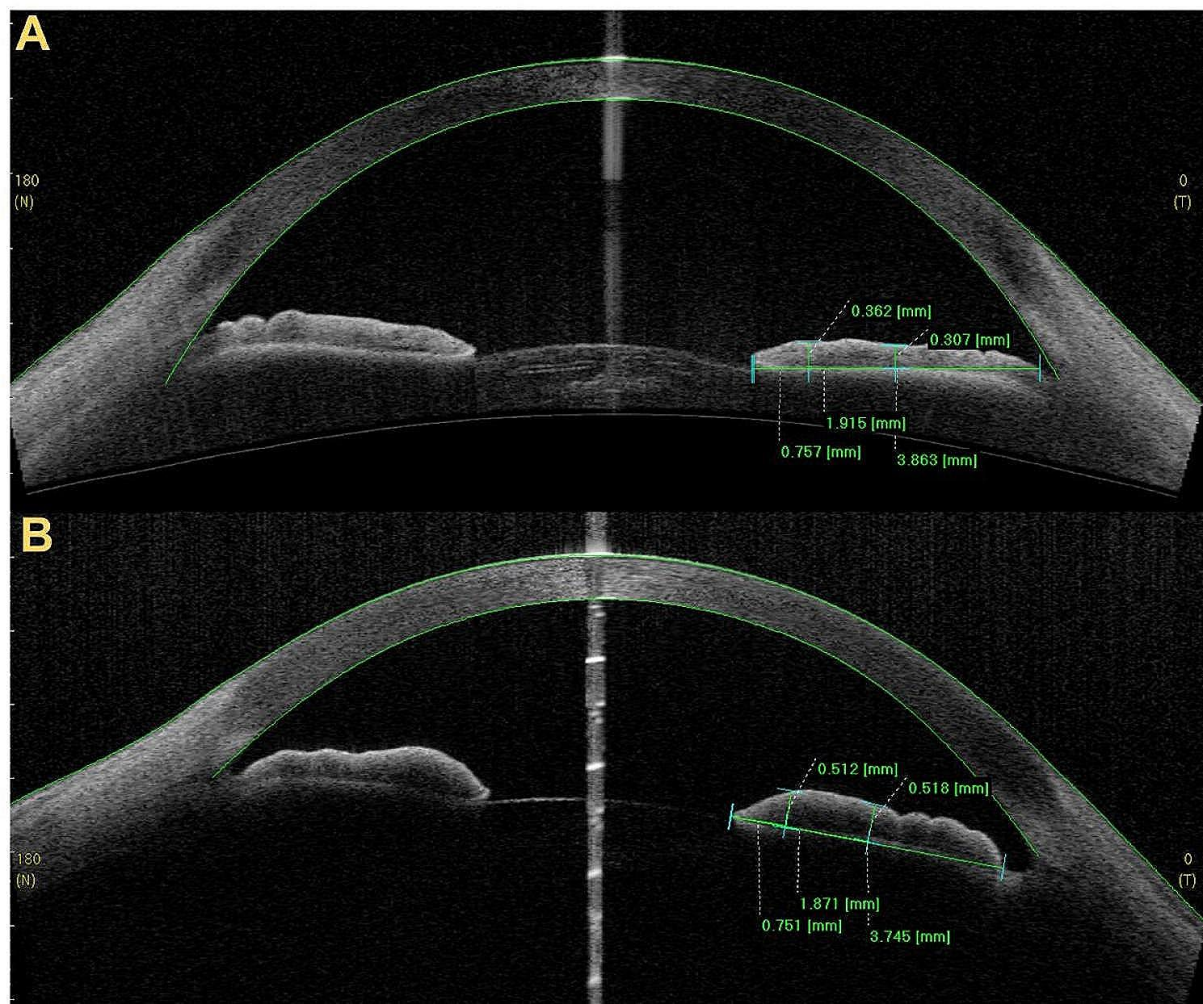
Picture **A** was an AS-OCT image of a type 2 diabetes adult, with an iris thickness at iris dilator muscle region of 0.307 mm. Picture **B** was an AS-OCT image of a healthy adult, with an iris thickness at iris dilator muscle region of 0.518 mm. The measurements for the iris sphincter muscle and the iris dilator muscle regions were taken at 0.750 mm from the pupillary margin and at the midpoint between the pupillary margin and the iris root, respectively. The procedure for these measurements involved first determining the distance from the pupillary margin to the iris root and marking the midpoint. Then, at the positions located 0.750 mm from the pupillary margin and halfway along the length of the iris from the pupillary margin, the iris thickness was measured perpendicular to the direction of the iris root.

This study adopted a unique method for the acquisition and quantification of iris OCTA images (Fig. 1). Image quality control was performed by both the Cirrus built-in signal strength system and manual scoring by two analyzers. Unqualified OCTA images were excluded. When analyzing the images, the temporal side was selected, and the area of the pupil to limbus was selected as the iris blood circulation index for the whole iris. The iris was divided into three parts from the pupil margin to the corneoscleral limbus; the one-third closest to the pupil edge was selected as the pupillary margin iris blood circulation index, and the remaining two-thirds were taken as the iris blood circulation index of the outer part. The blood flow indices used in this study included the following: [1] vessel area density (VAD) is a unitless ratio of the total image area occupied by the vasculature to the total image area in the binary vessel maps; an increase in VAD indicates an expansion of the blood flow area; and [2] vessel skeleton density (VSD) is the ratio of the length occupied by the blood vessels to the total area in the skeletonized vessel map; an increase in VSD indicates an increase in the total quantity of blood vessels. Considering the consistency of both eyes, the eye with higher quality iris OCTA and iris AS-OCT images was chosen to represent the individual’s characteristics. The calculation of iris volume was automatically performed by the built-in program in AS-OCT. The iris thickness at the iris sphincter and dilator regions were manually measured by two analysts using the built-in software in AS-OCT, and the average value was taken, with a distance of 0.75 mm from the pupil edge representing the thickness of the iris sphincter region and a distance of half the iris length representing the thickness of the iris dilator region (Fig. 2). This research method has been applied in the study by Prata and others [9].

The spherical equivalent is the sum of the spherical diopter and half the cylinder diopter. All data analysis was performed using SAS 9.4 (Cary, NC). When calculating the sample size, the paired difference formula in Statstodo (https://www.statstodo.com) was used, with α set to 0.05 and a test power of 80%. Setting the difference in pupil size after mydriasis between diabetic patients and healthy controls to 0.5 mm, with an SD of 0.70 and an acceptable error range of 0.1, a sample size of 32 individuals per group (two-tailed) was required to obtain a parameter estimate with a 95% confidence interval. The measurement data were expressed as the mean ± standard deviation, and the count data were expressed as frequency and percentage. The Kolmogorov‒Smirnov test was used to determine whether the measurement data were normally distributed. If the data were normally distributed, a t-test was used; otherwise, a Mann‒Whitney test was used. The statistical methods used in this study included t-tests, Pearson correlation analysis, and multiple regression analysis. A p value less than 0.05 was considered statistically significant.

## Results

### First section: pediatric type 1 diabetes patients

This study finally enrolled 34 children with type 1 diabetes and 36 healthy children as controls, and both groups met the sample size requirement. The disease duration in children with type 1 diabetes ranged from 1 year to 18 years, with an average value of 7.29 ± 3.93 years. There were 3 patients with a duration of diabetes of 0–2 years, 9 patients with a duration of diabetes of 3–5 years, and 22 patients with a duration of diabetes of more than 5 years. Glycated hemoglobin levels ranged from 5.5 to 12.3%, with a mean of 7.36% ± 1.49. The basic and ophthalmic examinations of all participants and the comparison between diabetic patients and healthy controls are shown in Table 1. There were no differences in age, sex, height, weight, blood pressure, axial length, refractive error spherical equivalent, or intraocular pressure between the two groups (*p* > 0.05).

**Table 1:** Basic and ophthalmic examinations of all participants

Parameters	Healthy Kids	T1DM Kids	*P* value	Healthy Adults	T2DM Adults	*P* value
	*N* = 36	*N* = 34		*N* = 50	*N* = 52	
**Best-corrected visual acuity (log MAR)**	-0.01 ± 0.03	-0.01 ± 0.04	0.450	0.43 ± 0.32	0.45 ± 0.40	0.331
**Age**	13.86 ± 2.37	14.38 ± 3.26	0.987	66.27 ± 8.18	68.16 ± 9.83	0.307
**Sex**	18Males,50%	17Males,50%	0.716	22Males, 44.0%	30Males, 57.7%	0.199
**Height**	157.76 ± 13.12	159.61 ± 13.15	0.731	1.65 ± 0.07	1.66 ± 0.09	0.577
**Weight**	51.92 ± 10.85	53.29 ± 13	0.239	65.44 ± 11.21	64.43 ± 12.51	0.735
**Systolic Pressure**	115.19 ± 11.00	116.73 ± 14.03	0.620	131.56 ± 18.21	134.35 ± 18.88	0.502
**Diastolic Pressure**	65.10 ± 10.75	67.48 ± 8.08	0.997	80.12 ± 12.90	75.43 ± 14.13	0.125
**Axial Length**	24.57 ± 0.91	24.62 ± 1.18	0.942	25.12 ± 2.08	24.36 ± 2.77	0.420
**Refractive Error Spherical Equivalent**	-3.08 ± 1.84	-2.63 ± 2.42	0.373	N/A	N/A	N/A
**Intraocular Pressure**	15.90 ± 2.32	17.02 ± 2.80	0.257	14.95 ± 3.40	14.85 ± 3.29	0.897

T1DM = type 1 diabetes mellitus; T2DM = type 2 diabetes mellitus

Since adult subjects had varying degrees of cataract and crystalline myopia, refractive error spherical equivalent was not collected

The diabetes duration of diabetic children was 7.29 ± 3.93 years, and the glycated hemoglobin level was 7.36 ± 1.4. The iris blood flow indices and morphological indices of the two groups of children at rest and after adequate pharmacological mydriasis are shown in Table 2. There were no significant differences in pupil diameter; VAD and VSD of the whole iris, outer two-thirds and the pupillary margin; iris thickness at the iris sphincter muscle region and the iris dilator muscle region; or iris volume before and after dilation among children with type 1 diabetes and healthy children. Multiple linear regression analysis of pupil diameter after drug-induced mydriasis in children with type 1 diabetes revealed no statistically significant influences on the baseline parameters in Tables 1 and 2 (not presented in the results).

**Table 2:** The changes of iris parameters after drug-induced mydriasis among type 1 diabetes mellitus children and healthy kids

	Parameters	Healthy Kids *N* = 36	T1DM Kids *N* = 34	*P* value
**Baseline**				
	**Pupil Diameter**	5.32 ± 0.85	5.14 ± 0.81	0.364
	**Whole VAD**	0.25 ± 0.07	0.23 ± 0.06	0.079
	**Whole VSD**	0.19 ± 0.05	0.17 ± 0.05	0.071
	**Outer 2/3VAD**	0.24 ± 0.06	0.23 ± 0.06	0.354
	**Outer 2/3VSD**	0.18 ± 0.05	0.17 ± 0.05	0.240
	**Pupillary Margin VAD**	0.30 ± 0.10	0.26 ± 0.07	0.109
	**Pupillary Margin VSD**	0.21 ± 0.07	0.19 ± 0.05	0.299
	**Iris Thickness at SMR**	0.41 ± 0.06	0.39 ± 0.07	0.632
	**Iris Thickness at DMR**	0.35 ± 0.06	0.34 ± 0.05	0.637
	**Iris volume**	33.35 ± 3.27	32.75 ± 4.10	0.502
**After Mydriasis**				
	**Pupil Diameter**	7.71 ± 0.61	7.69 ± 0.68	0.900
	**Whole VAD**	0.30 ± 0.08	0.28 ± 0.08	0.353
	**Whole VSD**	0.21 ± 0.05	0.21 ± 0.05	0.640
	**Outer 2/3VAD**	0.31 ± 0.07	0.29 ± 0.07	0.986
	**Outer 2/3VSD**	0.21 ± 0.07	0.20 ± 0.05	0.577
	**Pupillary Margin VAD**	0.22 ± 0.06	0.24 ± 0.09	0.361
	**Pupillary Margin VSD**	0.16 ± 0.07	0.18 ± 0.06	0.184
	**Iris volume**	28.22 ± 3.32	27.87 ± 4.18	0.706

T1DM = type 1 diabetes mellitus; VAD = vessel area density; VSD = vessel skeleton density; SMR = sphincter muscle region; DMR = dilator muscle region

### Second section: adult type 2 diabetes patients

Fifty adults with type 2 diabetes and 52 age-matched healthy adults were included in this study, and both groups met the sample size requirement. The duration of diabetes in adults ranged from 0.5 to 30 years with a mean of 9.12 ± 8.60 years, including 16 patients with 0–2 years, 12 patients with 3–5 years, and 24 patients with more than 5 years. Glycated hemoglobin levels ranged from 5.8 to 12.6%, with a mean of 7.78% ± 1.91. The basic and ophthalmic examinations of all participants and the comparison between diabetic patients and healthy controls are also shown in Table 1. There were no differences in age, sex, height, weight, blood pressure, axial length, refractive error spherical equivalent, or intraocular pressure between the two groups (*p* > 0.05).

The iris blood flow indices and morphological indices of the two groups at rest and after adequate drug-induced mydriasis are shown in Table 3. At baseline, the pupil diameter of type 2 diabetic adults was smaller than that of healthy adults (*p* = 0.050), the pupillary margin VSD was higher than that of healthy adults (*p* = 0.008), and the iris thickness at the iris dilator muscle region was thinner than that of healthy adults (*p* = 0.014). After pharmacological mydriasis, the pupil diameter in diabetic adults was significantly smaller than that in healthy adults (*p* = 0.011). Multiple linear regression analysis of pupil diameter in adults with type 2 diabetes mellitus after drug-induced mydriasis with the baseline parameters in Tables 1 and 3 revealed that glycated hemoglobin level was the only independent factor (β=-0.490, *p* = 0.031). The higher the glycated hemoglobin level was, the smaller the pupil diameter after drug-induced mydriasis.

**Table 3:** The changes of iris parameters after drug-induced mydriasis among type 2 diabetes mellitus adults and healthy adults

	Parameters	Healthy Adults *N* = 50	T2DM Adults *N* = 52	*P* value
**Baseline**				
	**Pupil Diameter**	4.77 ± 0.80	4.42 ± 0.84	0.050
	**Whole VAD**	0.21 ± 0.05	0.21 ± 0.06	0.550
	**Whole VSD**	0.15 ± 0.04	0.16 ± 0.04	0.095
	**Outer 2/3VAD**	0.20 ± 0.07	0.20 ± 0.07	0.786
	**Outer 2/3VSD**	0.15 ± 0.05	0.15 ± 0.05	0.854
	**Pupillary Margin VAD**	0.27 ± 0.06	0.29 ± 0.09	0.308
	**Pupillary Margin VSD**	0.18 ± 0.04	0.21 ± 0.05	0.008
	**Iris Thickness at SMR**	0.48 ± 0.11	0.45 ± 0.09	0.173
	**Iris Thickness at DMR**	0.50 ± 0.08	0.44 ± 0.11	0.014
	**Iris volume**	36.58 ± 4.28	35.22 ± 4.87	0.175
**After Mydriasis**				
	**Pupil Diameter**	7.58 ± 0.68	7.19 ± 0.68	0.011
	**Whole VAD**	0.30 ± 0.08	0.28 ± 0.10	0.337
	**Whole VSD**	0.21 ± 0.05	0.22 ± 0.06	0.829
	**Outer 2/3VAD**	0.29 ± 0.06	0.28 ± 0.07	0.655
	**Outer 2/3VSD**	0.21 ± 0.06	0.22 ± 0.06	0.763
	**Pupillary Margin VAD**	0.32 ± 0.08	0.30 ± 0.11	0.301
	**Pupillary Margin VSD**	0.22 ± 0.05	0.22 ± 0.06	0.736
	**Iris volume**	29.38 ± 3.97	29.44 ± 4.75	0.949

T2DM = type 2 diabetes mellitus; VAD = vessel area density; VSD = vessel skeleton density; SMR = sphincter muscle region; DMR = dilator muscle region

## Discussion

This study included diabetic patients without significant visual impairment or diabetic retinopathy, who can be considered relatively early-stage diabetes patients with mild ischemia and hypoxia in the eye. Therefore, the results of this study represent early iris changes in diabetic patients.

The pupil diameter of children with type 1 diabetes was slightly smaller than that of children in the control group at rest, but the difference was not statistically significant. This result is inconsistent with the previous study by Schwingshandl et al. [6] The possible reason for this is that the average age of the children with type 1 diabetes included in this study was younger, the disease course was shorter, and the degree of iris autonomic neuropathy was mild. Furthermore, the pupil diameters reported by Schwingshandl were measured under dark conditions, although the specific level of illumination was not detailed. In contrast, our measurements of static pupil diameter were conducted in an ambient environment. The illuminance of environment is known to affect pupil size, which could be one of the factors contributing to the differing results observed between our study and that of Schwingshandl. Additionally, in our study, there was a variation in the level of environmental illumination between the pediatric and adult participants, which could also be a potential reason for the observed differences in results across these two group of patients. In the eyes of adults with type 2 diabetes, the pupil diameter at rest was significantly smaller than that of the control group, which was consistent with the report by Jain [13] and others. In the past, many scholars have studied the microscopic changes in the iris smooth muscle of diabetic patients [8] and speculated that iris smooth muscle damage is an important cause of abnormal pupil diameter. However, there have been no studies on the relationship between iris smooth muscle and pupil dilation in patients in vivo. Although the iris sphincter muscle and dilator muscle antagonize each other under the dual control of the parasympathetic and sympathetic nerves, it is generally believed that the iris dilator muscle has a greater effect on pupil dilation. Previous studies by Prata et al. reported that there was significant thinning of the iris thickness at the iris dilator muscle area in patients who took α-adrenergic receptor blockers for an extended period of time, and they believed that this thinning was related to the weakening of the muscle strength and atrophy out of disuse of the pupil dilator muscle [9]. Inspired by Ritch’s research, this study also measured the iris thickness at the iris sphincter muscle and iris dilator muscle regions among the participants using the same method. The results showed that there was no difference between the iris thickness at the site of the iris sphincter muscle and iris dilator muscle regions in children with type 1 diabetes and healthy individuals, while the iris thickness at the iris dilator muscle region of adult type 2 diabetic patients was significantly thinner (Fig. 1), which is consistent with the results observed under the electron microscope in previous studies [8]. We believe that this may be because most children with type 1 diabetes included in this study had a short disease duration, and abnormal glucose metabolism had not yet led to severe iris smooth muscle damage, while most adults with type 2 diabetes had a longer disease course and had already undergone significant iris dilator muscle damage.

Diabetes damages small blood vessels through various mechanisms, such as producing oxidative stress and advanced glycosylation end products, which manifest in the eye as diabetic retinopathy and diabetic iridopathy [11]. In previous studies conducted by our study group, significant differences were also found between iris vessels in diabetic patients and healthy individuals. For instance, diabetic retinopathy patients showed a significant increase in the iris blood flow indices at the pupil margin before the onset of diabetic iridopathy or iris neovascularization [14]. The pupil margin is the region where the minor arterial circle is located, which contains numerous small and capillary vessels that are more susceptible to the effects of high blood glucose levels and where iris neovascularization often occurs first in diabetic patients [15]. In this study, our initial findings indicated no significant differences in the VAD and VSD across the various iris regions between children with type 1 diabetes with a short disease duration and healthy controls. However, we observed that the p-values for the overall VAD and VSD were approaching statistical significance. This observation suggests a potential trend that, with an increased sample size, might reveal statistically significant differences. If proven true, it would imply that children with short-duration T1DM exhibit lower whole iris VAD and VSD compared to their healthy counterparts of the same age, indicating early microvascular changes in the pediatric T1DM population. In type 2 diabetes patients, the pupillary margin VAD was similar to that of the control group, while the pupillary margin VSD was significantly greater than that of the control group (Fig. 2). Therefore, we hypothesize that in patients with a longer course of diabetes but without diabetic retinopathy, the small blood vessels at the pupil margin have already opened or developed new vessels, compensating for the ischemia caused by high glucose levels, thus ensuring local iris blood flow. Theoretically, if iris blood supply does not decrease, its nutritional effect on local iris smooth muscle and iris nerves can still be guaranteed. Therefore, we believe that among the adult patients with early diabetic eye diseases included in this study, iris blood circulation changes have not yet affected the iris dilator muscle of the pupil and iris nerves and thus have not indirectly promoted pupil abnormalities.

This study used different mydriatic eye drops in pediatric and adult diabetes patients, which is consistent with the clinical practice of selecting mydriatic agents for children and adults. Due to the difficulty in achieving paralysis of the ciliary muscle in children, a stronger M receptor blocker is needed. The two eye drops contained different ingredients; for pediatric patients, the pure M receptor blocker cyclopentolate was used, while for adult patients, a tropicamide compound containing both M receptor blockers and alpha receptor agonists was used. The two methods of administering the eye drops included passing through the corneal barrier into the aqueous humor and spreading in the iris and ciliary muscle and, rarely, being absorbed into the blood through the conjunctival sac or nasal mucosa and entering the eye and iris via the bloodstream [16]. In the latter case, iris blood vessels can affect the distribution of M receptor blockers and alpha receptor agonists in the iris, with diabetic patients who have good vascular function having higher local iris blood drug concentrations, and vice versa.

In this study, we found that in children with type 1 diabetes with a short disease duration, the pupil diameter after mydriatic drug use was essentially identical to that of patients in the control group, and no influencing factors related to the pupil diameter were found. We hypothesize that the observed results could be attributed to the degree of autonomic neuropathy in children with type 1 diabetes is lower, and the function of the iris smooth muscle is not different from that of healthy people. In diabetic adults, the pupil diameter after mydriatic drug use was significantly smaller than that of the control group, which is consistent with previous research results, and at this time, the difference in the pupillary margin VSD between the two groups was not statistically significant. Due to the reduction in iris volume after mydriasis, the vessel density increases significantly; diabetic patients have a smaller reduction in iris volume and a less obvious increase in vascular density than healthy people after mydriasis, resulting in the disappearance of the difference in VSD of the pupil edge between the two groups after mydriasis. In further linear regression analysis, it was found that the only independent factor of the pupil diameter after drug-induced mydriasis in adult type 2 diabetic patients was the level of glycated hemoglobin, which did not include the iris thickness at the sphincter pupillae or dilator pupillae at baseline or the quantitative index value of iris blood flow at baseline. As mentioned earlier, the changes in iris blood flow at baseline have not yet affected the iris dilator muscle and iris nerves. Although the iris dilator muscle has been damaged at baseline, it may not be severe. It is hypothesized that the degree of autonomic neuropathy in diabetes has a more direct and critical effect on the mydriatic pupil diameter. Due to the limitations of current detection technology, nerve damage of the iris cannot be directly observed in living eyes. Previous research in other tissues has confirmed that the degree of peripheral nerve damage in diabetic patients is directly related to blood glucose levels [17, 18]. The influence of glycated hemoglobin identified in this study’s regression analysis reflects the degree of damage to the iris’s autonomic nerves.

The shortcomings of this study are as follows: (1) The study subjects included only diabetes patients with mild ischemia and hypoxia in the early stage and mild iris lesions. (2) Due to the difficulty and inaccuracy in determining the smooth muscle area of the iris after dilation, the thickness at the iris sphincter and dilator muscle regions were not measured. (3) In our study, there is a notable disparity in gender distribution between the healthy adult group (44.0% male) and the adult T2DM group (57.69% male). While the statistical analysis did not reveal a significant difference (*P* = 0.199), this imbalance in gender proportions could influence results to some degree and may limit the generalizability of our findings. After searching the PubMed database, no previous quantitative study reports on the correlation between iris blood flow and pupil diameter were found in children with type 1 diabetes and adults with type 2 diabetes, and there were no studies on the iris thickness at the iris sphincter and dilator muscle regions in diabetic eyes. This study innovatively used iris sphincter and dilator muscle thickness measured by anterior segment optical coherence tomography (OCT) to study drug-induced mydriasis in diabetic patients.

## Conclusion

This study observed type 1 and type 2 diabetes patients who did not have significant visual impairment or diabetic retinopathy. In eyes without more advanced iris changes, children with type 1 diabetes with no significant changes in pupil diameter at rest also had no abnormalities in iris dilator muscle and iris blood flow. Type 2 diabetic adults with a significant decrease in pupil diameter at rest had thinner iris thickness in the iris dilator muscle region and greater iris vessel numbers at the pupil edge, but neither directly affected the pupil diameter after drug-induced mydriasis. The pupil diameter after drug-induced mydriasis was more related to diabetic autonomic neuropathy and glycated hemoglobin levels, which represented its degree of injury. In the future, we will continue to follow up and observe these patients to understand the new changes in the iris in diabetic eyes once significant ischemia and hypoxia occur. We will also further explore the relationship between iris blood flow changes and the development of autonomic neuropathy in diabetes, as well as the specific effects of iris blood flow on iris smooth muscles through animal models.

## Data Availability

All data generated or analysed during this study are included in this published article.

## Abbreviations

T1DM: Type 1 diabetes mellitus
T2DM: Type 2 diabetes mellitus
OCTA: Optical coherence tomography angiography
AS-OCT: Anterior segment optical coherence tomography
PD: Pupil diameter
SCADE: Shanghai Children and Adolescent Diabetes Eye
DR: Diabetes retinopathy
VAD: Vessel area density
VSD: Vessel skeleton density
SMR: Sphincter muscle region
DMR: Dilator muscle region
